# Family Support and Readiness to Consider Smoking Cessation among Chinese and Vietnamese American Male Smokers

**DOI:** 10.1155/2021/6678219

**Published:** 2021-05-08

**Authors:** Joan A. Daniel, Jin E. Kim-Mozeleski, Krishna C. Poudel, Angela Sun, Nancy J. Burke, Janice Y. Tsoh

**Affiliations:** ^1^College of Dental Medicine, University of New England, Portland, ME, USA; ^2^Prevention Research Center for Healthy Neighborhoods, Department of Population and Quantitative Health Sciences, Case Western Reserve University, Cleveland, OH, USA; ^3^Department of Health Promotion and Policy, School of Public Health and Health Sciences, University of Massachusetts Amherst, Amherst, MA, USA; ^4^Chinese Community Health Resource Center, San Francisco, CA, USA; ^5^Public Health, University of California Merced, Merced, CA, USA; ^6^Department of Psychiatry and Behavioral Sciences, University of California San Francisco, San Francisco, CA, USA

## Abstract

**Introduction:**

Smoking prevalence is disproportionately high among Asian American immigrant men with limited English proficiency. Understanding the role of family support may provide insights into culturally acceptable strategies to promote smoking cessation.

**Aims:**

This study examined how family support was associated with readiness to consider smoking cessation among Chinese and Vietnamese American male daily smokers.

**Methods:**

We analyzed baseline data (*N* = 340) from a cluster randomized trial of a family-based healthy lifestyle intervention. We assessed the frequency of receiving family support in various forms (encouraging use of cessation resources, praising efforts, checking in, and reminding of familial role). Multiple regression analysis was used to determine associations between family support areas and readiness to consider smoking cessation, controlling for covariates. *Results/Findings*. Reporting a higher frequency of receiving praise and encouragement for one's efforts to quit was positively associated with readiness to consider cessation. Other areas of family support were not significant.

**Conclusions:**

These findings provide evidence to explore specific areas of family support in enhancing Asian American smokers' readiness to consider cessation. As there is high interest from Asian American family members to support their smokers for quitting, culturally specific and acceptable strategies are needed to promote smoking cessation among Asian Americans.

## 1. Introduction

Cigarette smoking among Asian Americans is an important public health and health disparity issue. Although national studies estimate lower prevalence of smoking among Asian Americans compared to other racial/ethnic groups [1], specific subgroups of Asian Americans have high smoking prevalence. As smoking among men tends to be common across many Asian countries [2], Asian American men with lower levels of acculturation (e.g., immigrants with limited English proficiency) have disproportionately high smoking prevalence [2, 3]. In California, between 2014 and 2018, smoking prevalence was 9.1% among Asian men who spoke English very well, compared to 23.9% among counterparts with limited English proficiency [4].

There is a need to examine effective, culturally appropriate strategies to promote smoking cessation among Asian American immigrant men. East Asian cultures tend to emphasize interdependence [5], and existing research suggests that leveraging social support, particularly from family, is a promising strategy. For instance, in a study of Chinese and Vietnamese American current and former smokers, former smokers reported that receiving family encouragement for quitting was a key facilitator of the smoking cessation process [6]. Family members are also highly interested in supporting smokers to quit, evidenced partly by Asian language callers to the California Smokers' Quitline, of which 40% were “proxy” calls made by family or friends on behalf of smokers, compared to 6% of such calls to the English language helpline [7]. Qualitative findings from in-depth interviews with Chinese and Vietnamese American male smokers and their family members identified that familial and filial approaches (such as emphasis on quitting for the sake of the family's health) were strategies used to strengthen or reinforce smokers' commitments to quit [8]. Taken together, these prior findings suggest that identifying areas of family support for quitting could be a culturally congruent strategy for promoting smoking cessation in this population.

Leveraging family support for quitting can be more broadly conceptualized through social network theory, which posits that social networks impact health through social support, influence, engagement, direct contact, and access to resources [9]. A theoretical model by Westmaas and colleagues [10] posits that perceived or received social support influences motivation to quit as a precursor to cessation. However, findings on the role of social support in quitting have been mixed [11], partly because of heterogeneity of study designs, study populations, conceptualization of social support, and from whom support is received. Specific to Asian immigrant men, focus groups conducted with Vietnamese American male smokers and family members who participated in a family-based cessation intervention showed that increased family support for quitting enabled smokers to feel accountable to uphold their behavioral health goals [12].

The current study examined how Asian American male smokers' perceptions of family support for quitting were associated with their readiness to consider cessation (contemplation ladder). We examine data from Chinese and Vietnamese male daily smokers assessed prior to randomization in one of two family-based educational interventions. Our goal in conducting a secondary analysis of baseline data is to understand how areas of family support affect one's readiness to consider cessation in daily smokers who were motivated and unmotivated. Consistent with social network theory, we examined several domains of social support specific to family support for quitting.

## 2. Methods

The current study analyzed baseline data from the Healthy Family Project, a cluster randomized controlled trial examining the efficacy of a family-based smoking cessation intervention targeting limited-English-proficient Chinese and Vietnamese American male smokers and their nonsmoking family members (ClinicalTrials.gov registration NCT02307734). A recruitment strategy and eligibility criteria were described in published studies using the same data source [13–15]). Briefly, the study's lay health workers were trained to conduct participant recruitment (such as recruiting from their own social networks), and lay health workers recruited participants as dyads. Dyads consisted of a male daily smoker (defined as having smoked at least one cigarette daily in the past seven days, regardless of their current readiness to consider cessation) and a nonsmoking, household-residing family member of any gender. Eligibility criteria included age (aged 18 or older), ethnicity (self-identified Chinese or Vietnamese), and ability to speak and read Chinese or Vietnamese. Recruitment occurred between 2015 and 2017 in Northern California. This analysis examines survey data from 340 male daily smokers collected prior to randomization and any intervention activities through telephone surveys conducted by trained bilingual research staff. All study procedures were approved by the University of California San Francisco Institutional Review Board.

The outcome variable was readiness to consider cessation, assessed by the validated contemplation ladder [16]. Participants selected responses on a 5-point scale ranging from 0 to 4, with higher values reflecting greater readiness to consider cessation (see Table 1 for response statements) to the statement that “best describes where you are in your thinking about quitting smoking.”

Family support for quitting was assessed using four items, each intended to capture theoretically relevant forms of perceived support from family for quitting [17]. The domains were based on adaptations from measures of partner support for quitting (from the Partner Interaction Questionnaire [18] and the Support Provided Measure [19]) and qualitative research with Chinese and Vietnamese smokers and nonsmoking family members [8, 20]. The questions assessed how often in the last month the family member (a) encouraged use of cessation resources, (b) praised efforts, (c) checked in, and (d) reminded of familial role (see Table 1 for questions). Participants rated the frequency on a four-point scale ranging from 0 (never), 1 (rarely), 2 (sometimes) to 3 (very often). The variables were examined continuously and dichotomously (very often/sometimes vs. rarely/never).

Several demographic and smoking-related covariates that may be related to one's readiness to consider cessation were included. Demographic covariates included age, marital status, and education level, as prior studies have reported that these characteristics are associated with smoking in Asian Americans [21]. We included ethnicity (Chinese or Vietnamese), as an a priori covariate of the parent study, and participant self-rated health and baseline smoking characteristics, which included the number of years smoked regularly, average number of cigarettes per day, level of nicotine dependence measured through time to first cigarette after waking [22], and whether participants reported any past-year quit attempts.

Multiple regression analysis was used to examine the associations of family support variables with readiness to consider cessation. Each of the family support variables was entered as continuous variables. The analysis controlled for aforementioned demographic characteristics and smoking-related characteristics. Using PROC GENMOD in SAS, generalized estimating equations were used to account for clustering of participants by lay health workers, who conducted the participant recruitment and could recruit eligible participants from their own social networks. We computed the intraclass correlation (ICC) for the primary outcome variable (readiness to consider smoking cessation) using a variance component model in PROC GLM to estimate clustering.

## 3. Results

Table 1 displays participant characteristics and descriptive statistics. Mean readiness to consider cessation (0-4 scale) was 2.4 (SD = 1.4). Bivariate correlations between readiness to consider cessation and the family support variables ranged from 0.14 to 0.18 (all *p* values < 0.05; not shown on the table). Half of the participants (52.1%, *n* = 177) responded “rarely” or “never” to all four support questions, 19.1% (*n* = 65) reported receiving one support behavior (responding sometimes or very often), 14.4% (*n* = 49) reported two support behaviors, 10.0% (*n* = 34) reported three support behaviors, and 4.4% (*n* = 15) reported receiving all four support behaviors.

Table 2 displays results from the multiple regression analysis using generalized estimating equations examining family support and readiness to consider cessation, controlling for demographic characteristics and smoking-related covariates, and accounting for potential clustering effects. Only praising efforts to quit was associated with higher readiness, and the other areas of family support were not significantly associated. Participant ethnicity, past-year quit attempt, and the number of cigarettes smoked in a typical day were also significant in the analysis. Vietnamese smokers, compared to Chinese smokers, reported greater readiness to consider cessation at trial baseline. Reporting at least one past-year quit attempt was positively associated with readiness. The number of cigarettes per day was negatively associated with readiness. The ICC for readiness to consider cessation was 0.08.

## 4. Discussion

About one in six smokers (18%) reported receiving positive family support through praise and encouragement in their efforts to quit, and the frequency of praise and encouragement was associated with readiness to consider cessation, independent of smoking or demographic characteristics. The other forms of family support did not remain significant in the analysis, suggesting that it was specifically receiving positive, praise-oriented interaction that supported smokers' readiness to consider cessation. These results align with qualitative findings from focus groups that were conducted after a family-focused intervention, highlighting the impact of family member encouragement and social support for smoking cessation for Asian American male smokers [12].

Other variables significantly associated with readiness to consider cessation included past-year quit attempt, number of cigarettes smoked in a typical day, and ethnicity. Almost half reported at least one 24-hour quit attempt in the past year, which was associated with greater readiness to consider cessation. Participants who reported smoking more cigarettes were less ready to quit. Vietnamese participants reported higher readiness to consider cessation, even after adjusting for smoking characteristics. A prior study examining the role of tobacco use in influencing communication dynamics among Chinese and Vietnamese American immigrant dyads found that smoking directly contributed to family conflict and disrupted family harmony, leading to avoidance and noncommunication around tobacco use [20]. About half of the current participants reported no support-related interactions, suggesting noncommunication around tobacco use. Earlier work on smoking-related interactions among partners showed that the ratio of received positive versus negative support behaviors was associated with cessation [18]. Asian American smokers are aware of disruptions in family dynamics due to smoking [20], and so it is plausible that neutral behaviors (e.g., checking in) are perceived by the smokers as conflict-inducing or nagging. Further research is needed to understand ethnic differences found here.

Limitations to this study include inability to infer causality and possibility of recall bias. Smokers who were more motivated to quit may have perceived and/or received greater praise and encouragement from their family. As the measure of family support was specific to the household member who participated as a dyad, the provision of support from other family/household members could not be assessed. The analysis of baseline data from this trial allows for examining readiness to consider cessation prior to intervention and has implications for future research on provision of family support that matches participants' readiness.

Given the relatively high interest from Asian American family members to assist in the cessation process, this study examined several forms of family support and their association with readiness to consider cessation. These findings extend the literature by identifying a specific area of family support that can potentially be leveraged in future support-based intervention research to increase readiness to consider cessation and promote cessation. As Asian American male smokers with limited English proficiency continue to smoke at disproportionately high rates, acceptable culturally specific strategies are needed to promote smoking cessation in this group.

## Figures and Tables

**Table 1:** Sample characteristics of study participants (*N* = 340 male daily smokers).

Variables	*M* (SD) or %
Demographic characteristics and health status	
Age in years, *M* (SD)	54.6 (12.7)
Ethnicity	
Chinese	50.9%
Vietnamese	49.1%
Education level: graduated high school	55.9%
Marital status: married or living with a partner	88.2%
Self-rated health: excellent, very good, or good health	47.3%
Smoking characteristics	
# years smoked regularly, *M* (SD)	28.4 (14.3)
# cigarettes smoked in a typical day, *M* (SD)	9.5 (6.5)
Time to first cigarette after waking: 30 min or less^	48.5%
Quit attempt in the past year: made 1+ quit attempt	43.2%
Family support for quitting (0-3 scale)	
Encouraged use of cessation resources (“how often did your family member encourage you to use medication such as nicotine patch or nicotine gum, call the Asian Smokers' Quitline, or talk to your doctor to quit smoking?”), *M* (SD)	0.94 (1.17)
Very often or sometimes, %	14.7%
Praised efforts (“how often did your family member praise or encourage you for your efforts to quit smoking?”), *M* (SD)	1.13 (1.20)
Very often or sometimes, %	17.6%
Checked in (“how often did your family member ask you how things were going regarding your current smoking, or quitting situation?”), *M* (SD)	1.63 (1.13)
Very often or sometimes, %	26.5%
Reminded of familial role (“how often did your family member remind you that quitting will set a good example for your children or other family members?”), *M* (SD)	1.89 (1.11)
Very often or sometimes, %	36.5%
Readiness to consider cessation (contemplation ladder, 0-4 scale)	
I have not thought about quitting smoking, or I do not know (0)	13.8%
I think I need to consider quitting someday (1)	15.0%
I think I should quit but I am not quite ready (2)	20.3%
I am starting to think about how to change my smoking pattern (3)	22.4%
I am taking action to quit (4)	28.5%

Note: ^ does not include respondents who said “do not know”.

**Table 2:** Results from multiple regression analysis examining factors associated with readiness to consider smoking cessation.

Variables	*B* (SE)	*p*
Demographic characteristics and health status		
Age	0.01 (0.01)	0.23
Ethnicity: Vietnamese vs. Chinese	0.58 (0.16)	<0.001
Education level: graduated high school vs. less than high school	0.02 (0.16)	0.88
Marital status: married or living with a partner vs. not married	-0.19 (0.19)	0.30
Self-rated health: fair or poor vs. excellent, very good, or good	0.23 (0.14)	0.11
Smoking characteristics		
# years smoked regularly	-0.01 (0.01)	0.34
# cigarettes smoked in a typical day	-0.02 (0.01)	0.036
Time to first cigarette after waking: 30 min or less vs. 31 min or more	-0.16 (0.15)	0.29
Quit attempt in the past year: 1+ attempt vs. no attempts	0.39 (0.14)	0.006
Family support for quitting		
Encouraged use of cessation resources	0.22 (0.20)	0.28
Praised efforts	0.69 (0.20)	<0.001
Checked in	0.09 (0.22)	0.67
Reminded of familial role	0.23 (0.22)	0.30

Notes: regression analysis used generalized estimating equations to account for potential clustering effects of participant recruitment (conducted by lay health workers). Family support variables in the regression were continuous variables.

## Data Availability

The data used to support the findings of this study are available from the corresponding author upon request.
